# Supplementary material for: Experiences of Indigenous peoples living with pelvic health conditions: A scoping review
Source: PLoS One. 2025 Apr 3;20(4):e0307010. doi: 10.1371/journal.pone.0307010 (PMC11967981; doi:10.1371/journal.pone.0307010)
Supplement: Attachment — Submitted filename: Response to Reviewers-updated.docx [file pone.0307010.s005.docx]

20 December 2024

Dear Dr. Zahir and Reviewers,

Thank you for taking the time to review Experiences of Indigenous peoples living with pelvic health conditions: A scoping review, and for providing us with an opportunity to submit a revised manuscript. We appreciate the effort taken by the reviewers to provide in depth and valuable feedback. As outlined below, we have taken the suggested edits and incorporated them into the revised manuscript.

Reviewer 1:

1. Abstract
   a. Include Key Findings: Add a brief mention of the specific patterns or significant results found in the study. This will help readers quickly understand the main contributions of the review.
      i. See lines 26-33.
   b. Clarify Gaps: Specify the gaps identified in the literature more explicitly in the abstract, such as the underrepresentation of certain populations or regions.
      i. The patterns and gaps have been described more in the abstract – see lines 26-33.
2. Introduction
   a. Incorporate Recent References: Include more recent studies or reports (from the last 3-5 years) that highlight the importance of addressing cultural safety and trauma-informed care in Indigenous health research.
      i. We reviewed our citations and updated pelvic health and trauma-informed references to more recent publications.
      ii. We expanded more on cultural safety and trauma-informed practice in the methods section. See lines 133-154.
   b. Contextualize Indigenous Health Issues: Expand on the broader context of health disparities faced by Indigenous populations globally, linking these issues directly to the need for focused research on pelvic health conditions.
      i. An entire summary of health inequities experienced by Indigenous peoples is outside the scope of this manuscript. However, we included some additional wording on what contributes to health inequities, and what is currently known (or not known) about pelvic health conditions experienced by Indigenous peoples. See lines 45-53, and lines 63-75.
3. Methods
   a. Discuss Protocol Absence: Provide a more detailed discussion on the absence of a registered protocol, including potential impacts on the reproducibility and transparency of the review.
      i. See lines 80-85.
   b. Address Language Bias: Acknowledge the potential bias introduced by limiting the review to English-language publications. Discuss how this might have affected the inclusivity and comprehensiveness of the review.
      i. This is discussed in the Limitations section (see lines 478-484)
   c. Detail Inclusion/Exclusion Justifications: Provide a more thorough explanation of why certain studies were excluded, especially in the context of Indigenous research, where methodological approaches can be diverse.
      i. See lines 109-118.
4. Results
   a. Deeper Analysis of Gaps: Expand on the implications of the identified gaps, particularly the underrepresentation of specific regions (e.g., China, Russia, Nordic countries) and demographics (e.g., gender diverse populations).
      i. We believe the implications of the findings belong in the discussion section, see lines 381-383, 387-390 for more details.
   b. Clarify Exclusion Reasons: In the PRISMA flow diagram and text, clarify the reasons for excluding studies at different stages, especially during full-text screening.
      i. See lines 168-181, and Table 1.
   c. Enhance Data Presentation: Consider providing more granular data or sub-analyses where possible, such as comparing outcomes across different Indigenous groups or regions.
      i. We added in additional analysis: publications by year (lines 214-217, Fig 4), regional breakdown of interventional studies (lines 243-255, Fig 5), visualization of prevalence findings (Fig 7), and regional analysis of trauma-informed and culturally safe approaches (Safety heading, lines 233-241).
5. Discussion
   a. Link to Policy Implications: Strengthen the connection between the findings and potential policy implications, particularly how they could influence health service delivery and research funding for Indigenous populations.
      i. Thank you for pointing this out, especially for researchers that are not familiar with health policy and evidence-based decision making. See lines 418-428.
   b. Propose Practical Strategies: Offer specific, actionable strategies for incorporating cultural safety and trauma-informed approaches into future pelvic health research, such as recommended training for researchers or guidelines for ethical research practices.
      i. We added in some wording about what topics/training researchers should seek, and some resources to get them started (lines 447-453).
   c. Expand on Research Gaps: Discuss in more detail why certain conditions (e.g., autoimmune diseases, bowel conditions) are underreported and how future research could address these gaps.
      i. See lines 394-399 for discussion of gaps with respect to certain pelvic health conditions. Future research recommendations are discussed in lines 454-461.
6. Limitations
   a. Impact of Limitations: Provide a more in-depth discussion of how the limitations (e.g., language restrictions, lack of data from certain regions) might have influenced the study’s findings and conclusions.
      i. See lines 381-383 & 484-485 regarding regional representation, and lines 478-483 regarding language restrictions.
   b. Consider Alternative Approaches: Suggest alternative strategies that could mitigate the impact of the identified limitations, such as collaboration with researchers fluent in other languages or using broader search terms.
      i. As mentioned for question 3b, with respect to our language limits, see lines 478-483.
      ii. Our search terms were developed to be very broad, as evidenced by the number of abstracts (2911) compared to analyzed publications (242). We would be interested to learn what Reviewer 1 had in mind for increasing the breadth of the search.
7. Conclusion
   a. Strengthen Recommendations: Make the recommendations more forceful, particularly regarding the need for systemic changes in research practices and the importance of addressing identified gaps.
      i. We have provided more actionable recommendations for practice and research.
   b. Next Steps: Briefly outline the next steps for the authors' own research, including any planned studies or initiatives that will build on the findings of this review.
      i. See lines 496-499.
   c. Highlight Urgency: Emphasize the urgency of addressing the gaps identified, particularly in relation to improving health outcomes for Indigenous populations.
      i. From our perspective as non-Indigenous practitioners and researchers in women’s health, we agree that addressing these research gaps is important. However, Indigenous communities, governments, and organizations have differing priorities and concerns, and the research community should not be the ones to dictate which research topics are more urgent than others.
8. References
   a. Ensure Currency: Verify that all references are up-to-date, especially those related to recent developments in Indigenous health and pelvic health research.
      i. We have updated our references to include more recent publications relating to Indigenous health outcomes and trauma-informed practice.
   b. Balance of Sources: Ensure a balance between seminal works and recent studies, particularly in areas like cultural safety, trauma-informed care, and Indigenous health disparities.
      i. See above note.
9. Overall
   a. Engage Indigenous Communities: Emphasize the importance of engaging Indigenous communities in the research process, not just as subjects but as active participants in study design, implementation, and dissemination.
      i. Engagement and collaboration with Indigenous peoples is very important. We believe we have touched on the importance of this in lines 444-453.
   b. Highlight Ethical Considerations: Discuss the ethical considerations in conducting research with Indigenous populations, including obtaining informed consent, respecting cultural practices, and ensuring that the research benefits the community.
      i. We did not further elaborate on the ethical considerations of conducting research with Indigenous peoples, as we believe it falls outside the scope of this manuscript. Instead, we directed readers to several resources. See lines 450-453.
   c. Consider Visual Enhancements: Add more visual elements, such as tables or infographics, to present complex data more clearly and make the results section more engaging.
      i. Added three additional figures (Fig 4, Fig 5, Fig 7).

Reviewer #2:

1. Simplify the presentation of complex prevalence data to enhance accessibility for a broader audience.
   a. See Fig 7 for floating bar graph.
2. Consider adding brief explanations or visual aids to clarify the variability in reported rates.
   a. See Fig 7 for floating bar graph. Lines 272-273, and 277-278 provide a high-level overview of the prevalence findings, leaving the more detailed representation to the figure.

Reviewer #3:

1. The first paragraph of the introduction section should be moved to the end of the introduction, as it states the aim of the study, the lack of this subject in the literature, and its importance.
   a. See lines 68-75 for the recommended changes.
2. It was stated that “Search terms for pelvic health conditions were informed by the International Continence Society’s Incontinence, 6th Edition…”. We now have the 7th Edition. Therefore, it is suggested to check if the terms were informed by the 7th Edition and update this sentence if it refers to it.
   a. Search terms were developed in 2022, when the 6^th^ edition was the most recent version. We clarified this in the manuscript (see lines 94-96). We did not use the 7^th^ edition.
3. I suggest making some minor changes to figure 1 as I have some questions: (A) In line 151, it is written that the results were 2911, but it is 2894 in figure 1. Can the authors explain this discrepancy? I suggest mentioning at the bottom part of figure 1 that 40 publications were finally merged and a total of 203 publications were entered for analysis. (C) It is written that 20 full texts were included via citation searching. However, when we subtract 2032 (excluded abstracts) from 2422 (screened abstracts), we reach 390 full texts assessed for eligibility. What happened to those 20 full texts?
   a. Thanks for catching this error! Covidence’s PRISMA diagram was used to develop Fig 1, and the program sorts duplicates differently than how we presented it. Fig 1 has now been updated to be accurate. We also added more description in the text (see lines 168-181).
4. Were the Indian subcontinent and the Middle East excluded from Asia in figure 2? Please clarify this.
   a. Yes, see lines 186-188 for an explanation.
5. Were bladder control, bladder prolapse, and fistula excluded from the bladder conditions, and was bowel control excluded from the bowel conditions in figure 4? What conditions were labeled as bladder conditions and bowel conditions in this figure?
   a. See lines 261-269 for more detail of what was included in each category.
6. In line 229, it is stated that “Several publications described the influence of the healthcare system and its staff…”. How many were these publications in your review?
   a. Number of publications updated, see 287.

Formatting & Funding Disclosures

1. Please ensure that your manuscript meets PLOS ONE's style requirements, including those for file naming. The PLOS ONE style templates can be found at
   <https://journals.plos.org/plosone/s/file?id=wjVg/PLOSOne_formatting_sample_main_body.pdf> and 
   <https://journals.plos.org/plosone/s/file?id=ba62/PLOSOne_formatting_sample_title_authors_affiliations.pdf>
   a. Added indent to beginning of each paragraph.
   b. Modified Table 1 to align with formatting requirements.
   c. Removed author contributions and ORCID from title page.
   d. Removed bold formatting on supplemental material, figures, and table citations.
   e. Confirmed that font in the figures is Arial, size 8-12pt.
   f. Altered colours in figures to grey scale.
   g. Added legends to applicable figures (Fig 2-4).
   h. Reviewed reference list to comply with Vancouver style citations
2. Thank you for stating in your Funding Statement:
   "KB received funding support from the AbSPORU Graduate Studentship (<https://absporu.ca/>) while working on this study. EK was funded by the NWT-Network Environments for Indigenous Health Research (<https://cihr-irsc.gc.ca/e/51161.html>) as a reviewer. All other authors received no specific funding for this work." The funders had no role in study design, data collection and analysis, decision to publish, or preparation of the manuscript.

Please provide an amended statement that declares *all* the funding or sources of support (whether external or internal to your organization) received during this study, as detailed online in our guide for authors at <http://journals.plos.org/plosone/s/submit-now>.  Please also include the statement “There was no additional external funding received for this study.” in your updated Funding Statement. 
Please include your amended Funding Statement within your cover letter. We will change the online submission form on your behalf.

a. A revised funding statement is included at the end of our cover letter. I have confirmed with all co-authors about their funding, and have updated the statement.

Again, thank you for providing us with suggestions on how to improve this manuscript, and taking time out of your busy schedules to do so.

We look forward to hearing from you regarding this submission and to respond to any further questions and comments you may have.

Sincerely,

Kaeleigh Brown
